# The Efficacy of Eye Masks and Earplugs Interventions for Sleep Promotion in Critically Ill Patients: A Systematic Review and Meta-Analysis

**DOI:** 10.3389/fpsyt.2021.791342

**Published:** 2021-12-03

**Authors:** Leila Karimi, Farshid Rahimi-Bashar, Seyyede Momeneh Mohammadi, Mohsen Mollahadi, Masoum Khosh-Fetrat, Amir Vahedian-Azimi, Sara Ashtari

**Affiliations:** ^1^Behavioral Sciences Research Center, Life Style Institute, Nursing Faculty, Baqiyatallah University of Medical Sciences, Tehran, Iran; ^2^Department of Anesthesiology and Critical Care, School of Medicine, Hamadan University of Medical Sciences, Hamadan, Iran; ^3^Department of Anatomical Sciences, School of Medicine, Zanjan University of Medical Sciences, Zanjan, Iran; ^4^Exercise Physiology Research Center, Life Style Institute, Nursing Faculty, Baqiyatallah University of Medical Sciences, Tehran, Iran; ^5^Department of Anesthesiology and Critical Care, Khatamolanbia Hospital, Zahedan University of Medical Sciences, Zahedan, Iran; ^6^Trauma Research Center, Nursing Faculty, Baqiyatallah University of Medical Sciences, Tehran, Iran; ^7^Gastroenterology and Liver Diseases Research Center, Research Institute for Gastroenterology and Liver Diseases, Shahid Beheshti University of Medical Sciences, Tehran, Iran

**Keywords:** earplugs, eye mask, quality of sleep, intensive care unit, meta-analysis

## Abstract

Using physical devices such as eye masks and earplugs to improve to the quality of sleep in intensive care units (ICUs) is a very important issue. This study was conducted to assess the efficacy of eye masks and earplugs for sleep promotion in critically ill adult patients in the ICU based on various sleep quality assessment tools. PubMed, Scopus, Web of Science, and ProQuest were systematically retrieved until May 2021. Both randomized and non-randomized experimental and quasi-experimental studies were included if they evaluated the efficacy of eye masks and earplugs interventions on sleep outcomes in critically ill patients. The methodological quality was assessed by the Joanna Briggs Institute (JBI) critical appraisal tool. For the main outcome (sleep quality), a mean difference (MD) and confidence intervals (CIs) of 95% were determined. A total of 2,687 participants from 35 studies met the inclusion criteria. Twenty one studies were included in meta-analysis and 14 studies were included in the qualitative analysis. According to the results based on sleep quality assessment tools; overall scores of Pittsburgh Sleep Quality Index (PSQI) and Richards-Campbell Sleep Questionnaire (RCSQ), eye mask and/or earplug interventions have a positive effect on sleep quality. Based on Verran-Snyder-Halpern Sleep Scale (VSHSS), sleep disturbance was significantly lower in the intervention groups. In terms of polysomnography, the use of eye masks and/or earplugs resulted in a significant increase in total sleep time, sleep efficiency, rapid eye movement (REM) time, significant reduction of awaking, and sleep arousals index. The results of the present study suggest that the use of earplugs or eye masks, separately or combined affects sleep improvement in critically ill patients.

**Systematic Review Registration:** https://www.crd.york.ac.uk/prospero/display_record.php?RecordID=145830, PROSPERO: CRD42020145830.

## Introduction

Sleep in critical care settings was demonstrated to be of a poor quality, which is associated to both environmental-related factors (artificial light, ambient noise, alarms from monitoring devices, patient-care activities monitoring, diagnostic, and therapeutic procedures) and patients-related factors (old age, underlying diseases, pain, stress, psychosis, circadian rhythm disturbances, and organ dysfunction) (1, 2). Evidence suggests that the poor quality sleep in critically ill patients can cause both psychological and physiological consequences and also affect the recovery and treatment (3). Sleep disturbances may reduce immunodeficiency function, inspiratory muscle endurance, alter patients' weaning patterns, cardiorespiratory status, and increased pain scores in critically ill patients (4). In addition, it can leads to negative psychological states such as agitation, confusion and delirium (5, 6).

Sleep promotion interventions include both pharmacological and non-pharmacological treatments. Pharmacological agents that induce sleep provide sedation and analgesia and are commonly used in the ICU setting (7). However, pharmacological interventions can have negative side effects such as impaired cognitive function, the risk of tolerance or dependency, decreased ventilation, and a disruption in normal sleep physiology (8). Additionally, drug-induced sleep is contraindicated in certain patient groups, such as non-ventilated patients with hypercapnic lung disease (9). Therefore, today there is more emphasis and recommendation on non-pharmacological interventions. However, non-pharmacological interventions for improving sleep have been found to be less effective than pharmacological methods while posing no risk of drug-related tolerance or dependency (4, 10). Several non-pharmacological interventions including utilizing physical devices (eye masks and/or earplugs), relaxation techniques (massage and foot baths), music interventions, quiet time, acupuncture, and aromatherapy were attempt to improve to the quality of sleep in ICU (10).

Evidence shows that light and noise are the main cause of sleep disorders in the ICU (11, 12). Hence, it seems that the use of eye masks and earplugs as a low-cost intervention methods of noise reduction and light control can be superior to other interventions. Several studies found that the use of earplugs and eye masks improved sleep quality (13, 14). In addition, two systematic reviews by Alway et al. (15), and Locihova et al. (16), have highlighted benefits of earplugs and eye masks for improving sleep. But so far no meta-analysis has been done in this field. Therefore, we conducted this study to examine the efficacy of eye masks and earplugs for sleep promotion in critically ill patients based on various sleep quality assessment tools.

## Methods

### Search Strategy

This study was carried out in accordance with the Preferred Reporting Items for Systematic Review and Meta-Analysis (PRISMA) guidelines and recommendation by the Cochrane Collaboration for programming and conducting systematic reviews and meta-analyses (17, 18). Ethical approval was obtained from the research ethics committee of Baqiyatallah University of Medical Sciences with the ethics code of IR.BMSU.REC.1398.175. In addition, this systematic review has also been registered in international prospective register of systematic reviews (PROSPERO) with the registry code of CRD42020145830. Extensive electronic search was done in the following databases and search engines: PubMed, Scopus, Web of Science, and ProQuest. Combination of medical subject heading (Mesh terms) or synonyms, “eye masks,” “earplugs,” and “sleep” were used for carrying out literature search until May 2021 without restrictions in date and countries. Relevant articles in the reference lists of all included published articles were also searched manually. The full search strategy is available in Supplementary Material 1.

### Eligibility Criteria

Studies were eligible if they met all of the following inclusion criteria: (i) types of studies: randomized controlled trials (RCTs), randomized crossover studies, cluster randomized trials, and randomized or non-randomized quasi experimental (we included all studies, published or unpublished, in English and Persian language); (ii) types of participants: adult patients with stable hemodynamic condition who were admitted to ICUs, critical care units (CCUs), or in a simulated ICU conditions that is completely similar in terms of sound and light with no restrictions on gender or ethnicity; (iii) types of intervention: using eye mask and/or earplugs for improving sleep quality compare to routine standard care; (iv) outcome: the outcome measure sleep quality, which was measured by using standardized instruments including objective and/or subjective tools. Studies were excluded if they (i) enrolling participants who were diagnosed with obstructive sleep apnea or dementia or those who were terminally ill or required palliative care; (ii) conference articles, abstracts and protocols; (iii) examined a combination of other interventions (e.g., massage, foot baths, nursing interventions, valerian acupressure, and aromatherapy).

### Study Selection and Data Extraction

Two investigators (S.A, A.V-A) independently screened the full-text of the articles to select the studies satisfying the inclusion criteria. Then, the data and information were extracted according to the following study characteristics required for the current review; (a) general information: first author name, publication year and country; (b) method information: study design, study setting, study participants and sample size; (c) intervention: intervention type and assessment tools for sleep quality; (d) outcome: results of sleep quality. Any disagreements during this selection and extraction process were resolved either through consensus or consultation with third investigator (F.R-B).

### Risk of Bias Assessment in Included Studies

The quality assessment was performed by utilizing the Joanna Briggs Institute (JBI) critical appraisal tool (https://jbi.global/critical-appraisal-tools) for quasi-experimental and RCT studies, separately. Two reviewers independently assessed the risk of bias in each study. The RCT and quasi-experimental were evaluated based on 13 and nine criteria, respectively. All questions were answered as yes, no, not clear, or not applicable and assessed individually. Eligible studies were rated according to the dictionary and guidelines of the tool. After evaluating all the components of the study, the overall rating was determined using the criteria set out in the tool. Based on the number of “yes” responses, a rating of good = (≥10 yes), medium = (6–9 yes), and poor = (≤ 5 yes) was assigned to each RCT studies. For quasi-experimental studies, a rating of good = (≥7 yes), medium = (4–6), and poor = (≤ 3 yes) was assigned for nine questions (Supplementary Material 1).

### Statistical Analysis

The statistical analyses were conducted by STATA 16.0 (STATA Corp; College Station, Texas, USA) software. Included studies used different scales and instruments to measure sleep quality. Meta-analyses performed if outcomes from two or more studies with similar sleep quality assessment tool were available. To compare the use of earplugs or eye masks or both vs. no use of earplugs or eye masks, we used the mean difference (MD) with a 95% confidence interval (CI) for continuous data. Random effects models were performed to balance the effect quantity of each study. Statistical heterogeneity was assessed by *I*^2^, with *I*^2^ > 75% regarded as high heterogeneity. *P* < 0.05 was considered statistically significant. Moreover, to assess the publication bias, the Egger's (19) and Begg's (20) tests were conducted.

## Results

### Study Selection

**Figure 1:**
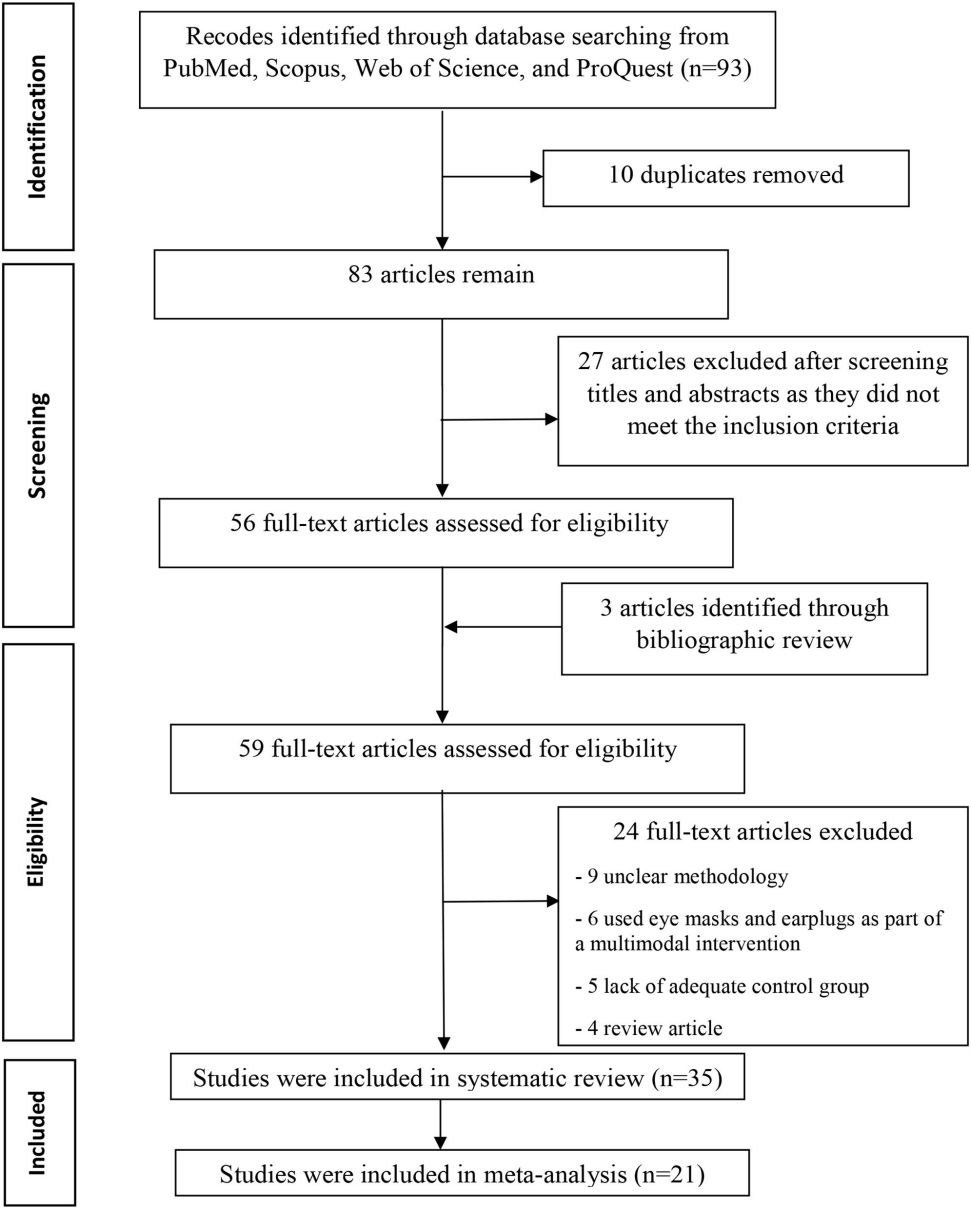
PRISMA (Preferred Reporting Items for Systematic Reviews and Meta-Analysis) flowchart detailing the disposition of screened, included, and excluded records.

The literature search results and the screening process are summarized in Figure 1. The search strategy yielded 93 records. A total of 37 records were excluded because they did not meet all predefined inclusion criteria or were duplicated. Moreover, we reviewed the bibliographies of the retrieved articles and found three more relevant studies. Fifty-nine full-text articles were evaluated for eligibility. Twenty-four studies were excluded due to unclear methodology, involve the use of eye masks and earplugs as part of a multimodal intervention, and lack of adequate control group. Thus, 35 full-text articles with 2,678 participants were included in the study. Meta-analyses were performed if outcomes from two or more studies with similar scales and sleep quality assessment tool were available. Therefore, 21 studied were included in the meta-analysis and the others (14 studies) were included in the qualitative analysis.

### Characteristics of the Studies Included

The characteristics of selected studies are presented in Table 1. The studies were conducted in USA (14, 21, 36, 48, 50, 51), UK (13, 22, 43), Belgium (23), China (24, 25, 27, 38), Iran (26, 28–32, 35, 39, 42, 49), France (33, 34), India (37, 41, 46), Australia (40), Jordan (44), Canada (45), Turkey (47), Egypt (52), and Singapore (53). Twenty eligible studies were RCTs (23, 24, 26, 28, 29, 33–35, 37–43, 46, 48, 51–53), six were randomized quasi-experimental (3, 21, 25, 27, 30), four were pre-post studies (5, 22, 31, 47), two were randomized cross-over studies (32, 49), and three were non-randomized quasi-experimental (13, 14, 45). Twenty-four trials were conducted in ICU which including medical ICU (5, 41, 46), cardiac ICU (13), general ICU (22, 33, 37, 44, 53), surgical ICU (32, 40, 48, 51), mixed medical and surgical ICU (23, 43), mixed medical and cardiac ICU (14), mixed medical and general ICU (42, 45, 50), neurology ICU (47), cardiac surgery intensive care unit (CSICU) (24, 52), and post-anesthesia care units (PACUs) (34). Nine trials were conducted in coronary care unit (CCU) (26–31, 35, 39, 49), and three trials were conducted in simulated ICU environment among healthy subjects (21, 25, 38).

**Table 1:** Characteristics and outcomes of all studies included.

**First author, year, country**	**Study type**	**Setting**	**Sample size**	**Intervention**	**Assessment tools for sleep quality**	**Outcomes measure**	**Conclusion**
Wallace, 1999, US (21)	Quasi-experimental randomized	Healthy persons in simulated ICU environment	Total: 6 I: 3 C: 3	Earplugs	PSG	REM latency (mean): 106.7 (SD:53.0) vs. 147.8 (53.0); *P* = 0.02 REM phase (mean: 19.9 (SD: 4.5) vs. 14.9 (5.4); *P* = 0.04	Positive effect on improved of REM, REM latency and sleep efficiency index
Richardson, 2007, UK (13)	Quasi-experimental Non-randomized	Cardiac ICU	Total: 62 I: 34 C: 28	Eye masks and earplugs	Original questionnaires created by authors	≥4 h sleep in intervention group: 15 (44.1%) ≥4 h sleep in control group: 10 (35.7%)	Improved quantity sleep in intervention group, no improvement in sleep quality
Scotto, 2009, US (14)	Quasi-experimental Non-randomized	Medical and cardiac ICU	Total: 88 I: 49 C: 39	Earplugs	VSHSS	Mean difference of sleep items score between two groups was (−3.253, *P* = 0.002)	Total sleep satisfaction scores were significantly better for the intervention group
Jones, 2008–2009, UK (22)	Pre-post study	General ICU	Total: 100 pre: 50 post: 50	Eye masks and earplugs	original questionnaires created by Richardson et al. (13)	≥4 h sleep in pre-intervention group: 23 (46%) ≥4 h sleep in post-intervention group: 24 (48%)	Patients reported sleeping for longer periods using earplugs and eye masks
Van Rompaey, 2008–2010, Belgium (23)	RCT	Medical and surgical ICU	Total:136 I: 69 C: 67	Earplugs	Original questionnaires created by authors	Sleeping with earplugs showed a significantly better sleep after the first night (*P* = 0.042)	Positive effects on sleep quality
Hu, 2009, China (24)	RCT	Cardiac Surgical ICU (CSICU)	Total: 45 I: 20 C:25	Eye masks and earplugs + relaxing music	RCSQ	Significant improved of subjective sleep quality and components in the intervention group	Positives effects of eye masks and earplugs on sleep quality
Hu, 2010, China (25)	Quasi-experimental randomized	Healthy persons in simulated ICU environment	Total: 14 I: 7 C: 7	Eye masks and earplugs	PSG	Improved REM sleep, shorter REM latency, and fewer arousals, (*P* < 0.05)	Positives effects of eye masks and earplugs on sleep quality
Daneshmandi, 2010, Iran (26)	RCT	Coronary care unit (CCU)	Total: 60 I: 30 C: 30	Eye masks	PSQI	Mean score of overall PSQI after intervention in test and control group was (4.86 ± 1.88 and 8.43 ± 1.97; *P* < 0.05)	Significant improved of subjective sleep quality and components in the intervention group
Ryu, 2010, China (27)	Quasi-experimental randomized	Coronary care unit (CCU)	Total: 58 I: 29 C:29	Eye masks and earplugs with relaxing music	VSHSS	Sleeping quantity: (279.3 ± 43.9 vs. 243.1 ± 42.6, *P* = 0.002 Sleep quality (36.1 ± 5.6 vs. 29.4 ± 3.8, *P* < 0.001) between groups	Sleep-inducing music significantly improved sleep quality in patients
Nieseh, 2010, Iran (28)	RCT	Coronary care unit (CCU)	Total: 60 I: 30 C: 30	Eye masks and earplugs	PSQI	Significant differences in PSQI was observed after intervention between groups (experimental group 6 ± 2.3, control group 8.8 ± 2.4 (*p* < 0.05)	Using the ear and eye protect device significantly improved sleep quality
Neyse, 2011, Iran (29)	RCT	Coronary care unit (CCU)	Total: 60 I: 30 C: 30	Earplugs	PSQI	Significant differences in PSQI was observed after intervention between groups (experimental group 6.3 ± 2.1, control group 8.4 ± 1.9 (*p* < 0.05)	Using earplugs can improve sleep quality in patients
Baghaei, 2011–2012, Iran (30)	Quasi-experimental randomized	Coronary care unit (CCU)	Total: 40 I: 20 C: 20	Eye masks	Leeds sleep evaluation questionnaire (LSEQ)	After intervention, the average total sleep score in control group was 4.8 ± 0.5, while in the eye mask group it was 6.7 ± 1.1 (*P* < 0.001)	Using of eye mask improves sleep quality in patients hospitalized in intensive cardiac care units
Mashayekhi, 2012, Iran (31)	Pre and post design	Coronary care unit (CCU)	Total: 60 I: 30 C: 30	Eye masks	VSHSS	In sub scale “effectiveness,” mean score of sleep quality was 255.33 ± 41.1 before intervention and 291.50 ± 38.9 after intervention	Using eye mask have statistically significant increased the quality of sleep in subscales disturbance and effectiveness
Yazdannik, 2012, Iran (32)	cross-over RCT	Surgery ICU	Total: 50 I: 25 C: 25	Eye masks and earplugs	VSHSS	Significant positive effects on sleep disturbance (*P* < 0.001) sleep supplementation (*P* < 0.01) sleep effectiveness (*P* = 0.03)	Using of eye mask improves sleep quality in patients
Demoule, 2011–2013, France (33)	RCT	General ICU	Total: 51 I: 23 C: 28	Eye masks and earplugs	PSG	- Prolonged awakenings were less frequent in the intervention group (21 vs. 31, *P* = 0.02)	No significant difference was observed between two groups in terms of sleep quality
Guen, 2013, France, (34)	RCT	Post-anesthesia care units (PACUs)	Total: 41 I: 20 C: 21	Eye masks and earplugs	Medical Outcome Study Scale (MOSS) and the Spiegel Scale (SS)	In the intervention group, sleep disruptions evaluated with the MOSS scale were fewer [4 (1–7) vs. 7 (3–10), *p* < 0.05]	Using of eye mask improves sleep quality in patients
Babaii, 2013 Iran (35)	RCT	Coronary care unit (CCU)	Total: 60 I: 30 C: 30	Eye masks	PSQI	Median (IQR) score of overall PSQI after intervention in the experimental group were significantly lower than those in the control group [3 (5–2) vs. 10 (12–7), *P* < 0.05]	Using of eye mask improves sleep quality in patients
Kamdar, 2013, US (36)	Pre-post test study	Medical ICU	Total: 300 I: 110 C: 185	Earplugs	RCSQ	The use of earplugs and eye masks significant improved sleep quality *P* = 0.02	Improvement quality of sleep
Bajwa, 2014, India (37)	RCT	General ICU	Total: 100 I: 50 C: 50	Eye masks and earplugs	VSHSS	sleep fragmentation (14.6 ± 3.44 vs. 4.19 ± 3.58), sleep latency (6.05 ± 1.88 vs. 1.70 ± 1.66), sleep quality (10.5 ± 2.52 vs. 2.14 ± 2.29), sleep length (8.95 ± 2.47 vs. 2.36 ± 2.46), sleep supplementation (11.8 ± 3.26 vs. 4.10 ± 2.33) in intervention and control groups, respectively	Improvement quality of sleep
Huang, 2014, China (38)	RCT	Healthy persons in simulated ICU environment	Total: 40 I: 20 C: 20	Eye masks and earplugs	PSG	Less awakenings and shorter sleep onset latency in the intervention group (*P* < 0.05)	Improvement quality of sleep
Cheraghi, 2104, Iran (39)	RCT	Coronary care unit (CCU)	Total: 72 I: 36 C: 36	Earplugs	PSQI	The mean ± SD of quality of sleep for the intervention group using earplugs decreased from 8.11 ± 3.00 (before the intervention) to 6.00 ± 2.30 (after the intervention). It increased from 6.33 ± 3.08 to 8.80 ± 2.45 for the control group (*P* = 0.001)	Using of earplugs improves sleep quality in patients hospitalized in intensive cardiac care units
Litton, 2015–2016, Australia (40)	RCT	Surgery ICU	Total: 40 I: 20 C: 20	Earplugs	RCSQ	The median RCSQ sleep summary scores were 43 (IQR, 20–58) and 45 (IQR, 29–64) for the earplugs and no earplugs groups, respectively (median difference, 2; 95% CI,−21 to −25; *P* = 0.58)	No significant difference was observed between two groups in terms of sleep based on RCSQ
Chaudhary, 2016, India (41)	RCT	Medical ICU	Total: 60 I: 30 C: 30	Eye masks and earplugs	Original questionnaires created by authors	The sleep quality score was improved after the administration of earplugs and eye mask among both the groups (*P* < 0.001)	Improvement quality of sleep
Sharafi, 2016, Iran (42)	RCT	General and medical ICU	Total: 73 I: 36 C: 37	Eye masks and earplugs	VSHSS	Sleep quality score in intervention group and control group were 45.41 ± 3.78 and 45.45 ± 5.61, respectively.	No significant difference was observed between the groups
Sweity, 2017, UK (43)	RCT	Medical and surgical wards	Total: 206 I: 109 C: 97	Eye masks and earplugs	Original questionnaires created by authors	Sleep quality was significantly higher in intervention group, (5.09 ± 2.05 vs. 6.33 ± 2.13, mean difference was 1.24, *P* < 0.001)	Improvement quality of sleep
Bani Younis, 2017, Jordan, (44)	Quasi-experimental Randomized	General ICU at 2 Hospital	Total: 103 I: 52 C: 51	Eye masks and earplugs	RCSQ	The mean RCSQ scores were (47.2 ± 16.5 vs. 36.2 ± 15.1, *P* < 0.001) for the intervention and control groups, respectively	Improvement quality of sleep
Dobing, 2017, Canada, (45)	Quasi-experimental Non-randomized	General and medical	Total: 81 I: 40 C: 41	Eye masks and earplugs	VSHSS	Sleep disturbance (median 420 vs. 359, *p* = 0.19), efficacy (median 169 vs. 192, *p* = 0.29), and supplementation (median 97 vs. 100, *p* = 0.51) scales were not significant difference between groups	No significant difference was observed between the groups
Arttawejkul, 2017–2018, India (46)	RCT	Medical ICU	Total: 17 I: 8 C: 9	Eye masks and earplugs	PSG and RCSQ	Polysomnographic parameters including total sleep time, sleep efficiency, wake after sleep onset, sleep latency, % rapid eye movement (REM) sleep, and % N3 sleep were similar between two groups (*P* > 0.05)	Based on PSG sleep quality domains were similar between groups and subjective sleep quality according to RCSQ score did not demonstrate the difference between the groups
Koçak, 2017–2018, Turkey (47)	Quasi-experimental non-randomized	Neurology ICU	Total: 64 I: 32 C: 32	Eye masks and earplugs	RCSQ	The RCSQ mean (SD) pretest and posttest scores were 50.21 (16.02) and 68.50 (17.57), respectively, for the experimental group and 55.34 (16.62) and 49.03 (15.53), respectively, for the control group	Improvement quality of sleep
Obanor, 2018, US (48)	RCT	Surgical ICU	Total: 23 I: 12 C: 11	Eye mask s and earplugs	RCS)	Postoperative days 1 and 2 respectively, aggregate mean RCSQ scores were (29.42 ± 25 and 38.33 ± 25) in the control group (*n* = 9) vs. (54.77 ± 23) and (65.22 ± 24) in the intervention group (*n* = 14)	Improvement quality of sleep
Baghaie Lakeh, 2018, Iran (49)	Cross-over RCT	Coronary care unit (CCU)	Total: 96 I: 48 C: 48	Earplugs	VSHSS	In the first night; the use of earplugs significantly reduced the quality of sleep disturbance domain in both groups A and B (*P* = 0.0001 and *P* = 0.021, respectively), and the supplementary sleep domain in group A (*P* = 0.027).	No significant difference was observed between the groups
Ho, 2018–2019, US (50)	Non-Randomized Control Trial	General medical	Total: 215 I: 109 C: 106	Eye masks and earplugs	Insomnia severity index (ISI) questionnaire	No significant adjusted OR in terms of insomnia (OR: 0.8, 95% CI: 0.34–1.87, *p* = 0.61) Satisfaction score: (4.22 ± 1.08 vs. 4.36 ± 0.86, *p* > 0.05) duration of stay: (5.14 ± 6.75 vs. 5.47 ± 6.08, *p* > 0.05)	No significant difference was observed between the groups
Obanor, 2018–2019, US (51)	RCT	Surgical ICU	Total: 87 I: 44 C: 43	Eye masks and earplugs	RCSQ	Compared with the control group's average RCSQ total score of 47.3 (95% CI, 40.8–53.8), the intervention group's average RCSQ total score was significantly higher at 64.5 (95% CI, 58.3–70.7; *P* = 0.0007)	Improvement quality of sleep
Mahran, 2107, Eygept (52)	RCT	Cardiac surgery intensive care unit (CSICU)	Total: 66 I: 31 C: 35	Eye masks	RCSQ	A statistically significant difference was found between groups in mean total RCSQ score over the 3-day study period (*P* = 0.001), with the intervention group reporting better sleep quality	Improvement quality of sleep
Leong, 2018–2019, Singapore (53)	RCT	General ICU	Total: 93 I: 48 C: 45	Eye masks and earplugs	RCSQ	Median [IQR (range)] sleep scores were 64 [38–74 (0–100)] and 60 [44–82 (18−100)] for the control and intervention groups, respectively (*P* = 0.310)	No significant difference was observed between the groups

*I, Intervention group; C, Control group; PSG, Polysomnography; RCSQ, Richards-Campbell Sleep Questionnaire; VSHSS, Verran-Snyder-Halpern Sleep Scale; PSQI, Petersburg's Sleep Quality Index*.

Several sleep assessment tools have been used in the reviewed studies. The majority of the publications used subjective tools, while only five of them employed the form of objective evaluation (21, 25, 33, 38, 46). Polysomnography (PSG) was the only form of objective method of assessment that used in these studies. Among the subjective tools for sleep evaluation, the Richards- Campbell Sleep Questionnaire (RCSQ) (5, 24, 44, 46–48, 51–54), and the Verran and Snyder-Halpern Sleep Scale (VSHSS) (14, 27, 31, 32, 37, 42, 45, 49) were the most frequently used in 11 and eight studies, respectively. Five trials used Pittsburgh sleep quality index (PSQI) as sleep assessment tool (26, 28, 29, 35, 39). Five studies (13, 22, 23, 41, 43), used their original sleep questionnaires. In addition, three studies used variable assessment tools such as Medical Outcomes Study Sleep (MOSS) score (34), Insomnia Severity Index (ISI) questionnaire (50), and Leeds Sleep Evaluation Questionnaire (LSEQ) (30).

### Sleep Quality Outcomes Based on PSQI

**Figure 2:**
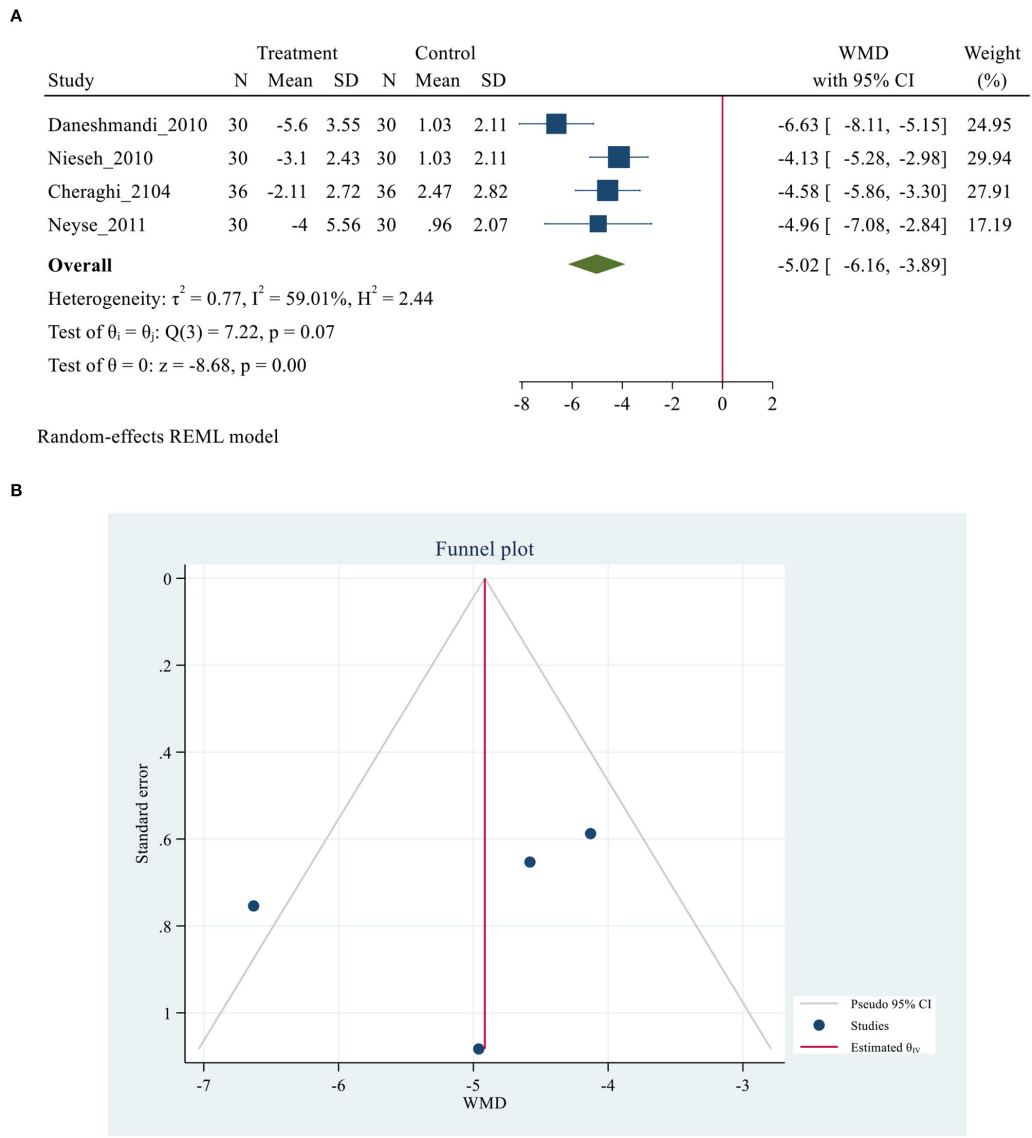
**(A)** Forest plot of mean difference (MD) for sleep quality based on PSQI between intervention and control groups. **(B)** Funnel plot showing publication bias on PSQI -based sleep quality.

Five studies (26, 28, 29, 35, 39) with 312 participants (156 patients in each control and treatment group), reported data on sleep outcomes using the PSQI scale. A study by Babaii et al. (35), reported the overall scale of PSQI *via* median (IQR), while the others reported by mean (SD). Thus, the meta-analysis was performed in the four studies (26, 28, 29, 39), and the qualitative analysis was conducted in the latter one study because the data of them could not be combined. A total PSQI score ranges from 0 to 21. A higher score suggests worse overall sleep quality, and a total cut-off PSQI score < 5 indicates good sleep (55). The meta-analysis of combined data conducted, showed a positive effect of used eye masks and/ or earplugs interventions on overall sleep quality based on PSQI score (MD= −5.02, 95% CI = −6.16 to −3.89, *P* < 0.001), with substantially heterogeneity among the studies (*I*^2^ = 59.01%, *P* = 0.07) (Figure 2A). The result revealed that the average PSQI score of the eye masks and/ or earplugs group was 5.02 points lower than that of the control group and indicating that the interventions might be beneficial to improve overall sleep quality. *P*-values of Egger and Begg tests indicated non-significant coefficient values for publication bias (Egger test: *P* = 0.635 and Begg test: *P* = 0.065) (Figure 2B). The PSQI scale consists of seven components including subjective sleep quality, sleep latency, sleep duration, habitual sleep efficiency, sleep disturbance, use of sleeping medications, and daytime dysfunction. Three studies (26, 28, 29), provided the data on the PSQI components, thus the meta-analyses were conducted to explore the efficacy of eye masks and/or earplugs interventions on sleep components, as shown in Table 2. No significant results were obtained for sleep components based on PSQI score (*P* > 0.05) (Supplementary Figures 1–3).

**Table 2:** Efficacy of eye masks and/or earplugs interventions for sleep components based on PSQI.

**Sleep components**	**Pooled MD (95% CI)**	***P*-value**	***I*^2^ (%)**	**Egger *P*-value**	**Begg *P*-value**
Sleep quality	−0.12 (−0.32, 0.09)	0.26	31.3	0.225	0.292
Sleep latency	−0.24 (−0.55, 0.07)	0.13	70.8	0.065	0.055
Sleep duration	−0.12 (−0.35, 0.11)	0.30	60.5	0.377	0.500
Habitual sleep efficiency	−0.11 (−0.38, 0.15)	0.40	59.9	0.067	0.296
Sleep disturbance	−0.34 (−0.81, 0.13)	0.16	90.2	0.060	0.500
Use of sleeping medications	−0.05 (−0.83, 0.73)	0.90	95.5	0.085	0.500
Daytime dysfunction	−0.51 (−1.21, 0.18)	0.15	94.1	0.052	0.296

*MD, Mean Difference; CI, Confidence Interval*.

### Sleep Quality Outcomes Based on RCSQ

**Figure 3:**
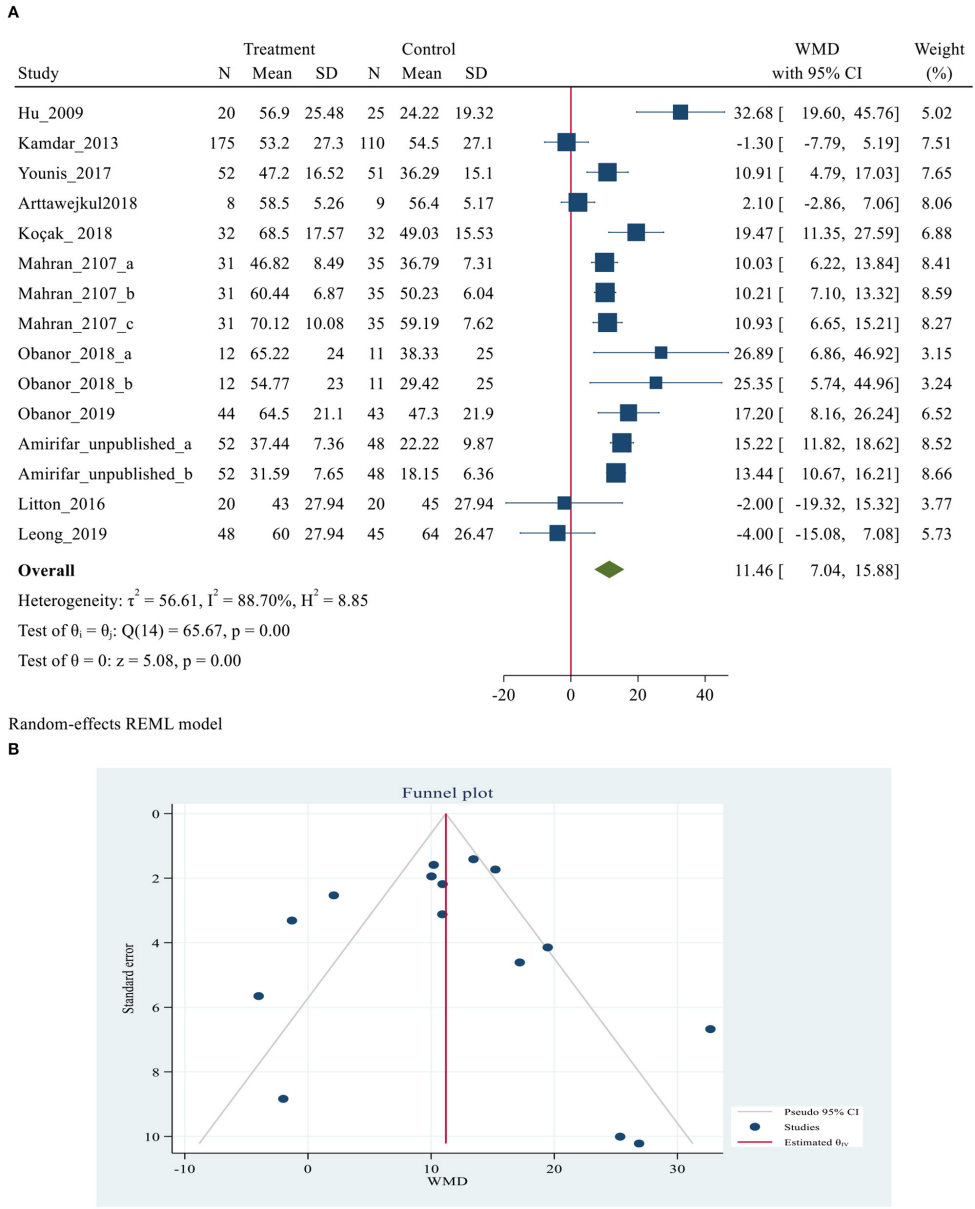
**(A)** Forest plot of mean difference (MD) for sleep quality based on RCSQ between intervention and control groups. **(B)** Funnel plot showing publication bias on RCSQ -based sleep quality.

Ten studies (3, 5, 24, 40, 46–48, 51–53), with 1,078 participants (568 and 510 patients in the intervention and control group, respectively), reported data on sleep outcomes using the RCSQ scale. RCSQ responses were graded on a 0–100 mm visual analog scale, with higher scores indicating better sleep. A score of 0–25 indicates poor sleep, while a score of 76–100 indicates good sleep (56). The RCSQ mean score of 11 studies for intervention groups was significantly higher than the mean score of the control groups (55.01 ± 15.43 vs. 40.15 ± 14.71, *P* = 0.007). The meta-analysis demonstrated a positive effect of using eye masks and/or earplugs on overall sleep quality based on RCSQ (MD = 11.46, 95% CI = 7.04–15.88, *P* < 0.001). However, substantial heterogeneity was also observed across the studies (*I*^2^ = 88.70%. *P* < 0.001). The results showing that the average RCSQ score of the treatment group was 11.46 points higher than that of the control group and indicating that the intervention might be beneficial to improve overall sleep quality based on RCSQ score in critically ill patients (Figure 3A). *P*-values of Egger and Begg tests indicated non-significant coefficient values for publication bias (Egger test: *P* = 0.269 and Begg test: *P* = 0.692) (Figure 3B). The RCSQ is a 5-item questionnaire that is used to assess sleep depth, latency, number of awakenings, efficiency, and sleep quality. Five studies (3, 24, 40, 48, 52), provided the data on the five-subdomain of RCSQ, thus the meta-analyses were conducted to explore the efficacy of eye masks and/or earplugs interventions on sleep subdomains based on RCSQ, as shown in Table 3. Significant results were obtained for all subdomains; sleep depth (MD = 9.88, 95% CI = 7.97–11.80, *P* < 0.001), sleep latency (MD = 13.17, 95% CI = 7.45–18.9, *P* < 0.001), number of awakenings (MD = 10.87, 95% CI = 8.90–12.84, *P* < 0.001), sleep efficiency (MD = 15.36, 95% CI = 7.27–23.46, *P* < 0.001), and sleep quality (MD = 12.59, 95% CI = 6.50–18.68, *P* < 0.001) (Supplementary Figures 4–6).

**Table 3:** Efficacy of eye masks and/or earplugs interventions for sleep components based on RCSQ.

**Sleep components**	**Pooled MD (95% CI)**	***P*-value**	***I*^2^ (%)**	**Egger *P*-value**	**Begg *P*-value**
Sleep depth	9.88 (7.91, 11.8)	<0.001^*^	0	0.357	0.368
Sleep latency	13.17 (7.45, 18.9)	<0.001^*^	82.1	0.060	0.368
Number of awakening	10.87 (8.9, 12.84)	<0.001^*^	0	0.799	0.368
Sleep efficiency	15.36 (7.27, 23.4)	<0.001^*^	91.1	0.561	0.368
Sleep quality	12.59 (6.5, 18.68)	<0.001^*^	82.7	0.386	0.368

* *P < 0.05 was considered as significant*.

*MD, Mean Difference; CI, Confidence Interval*.

#### Sleep Quality Outcomes Based on VSHSS

**Figure 4:**
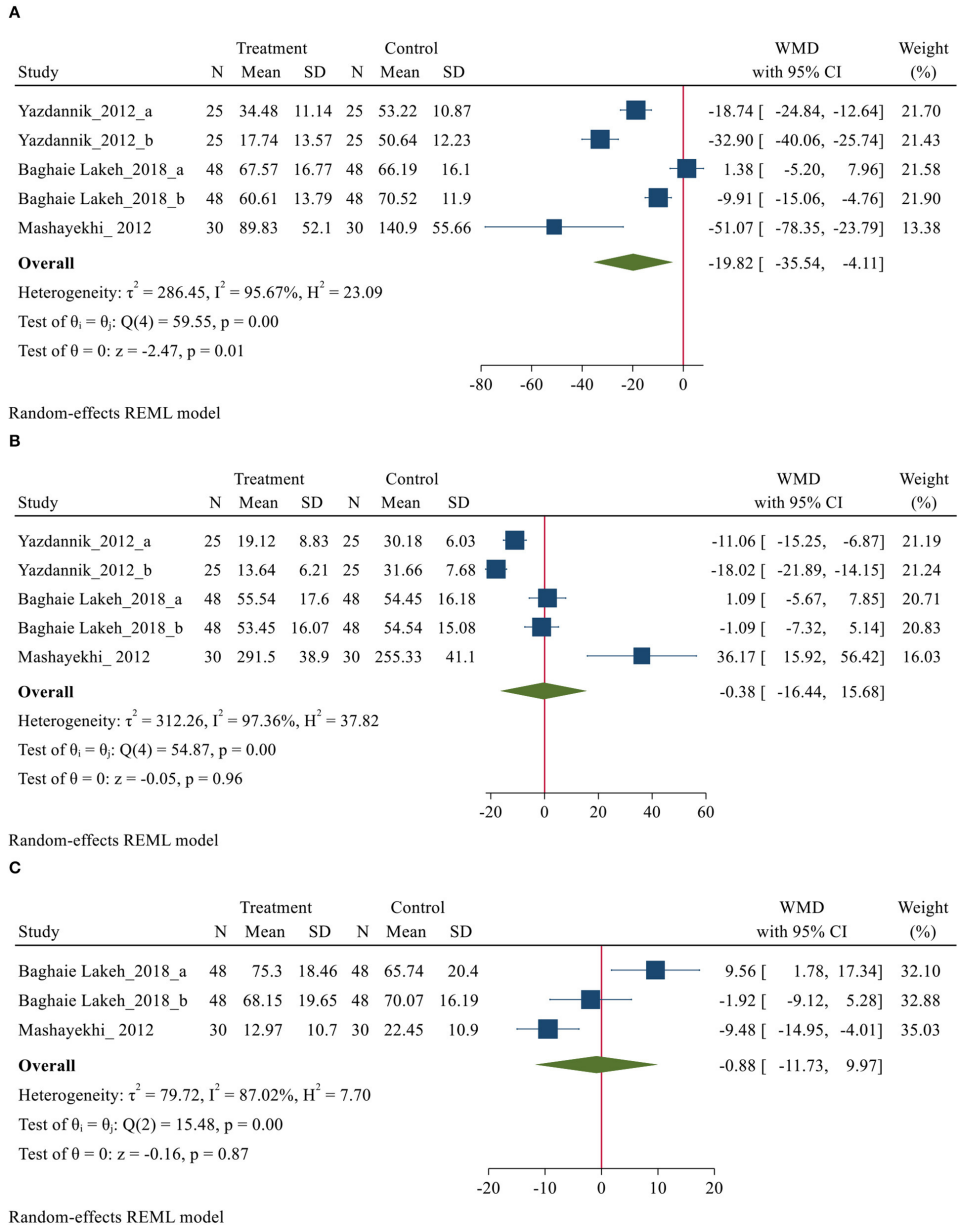
Forest plot of mean difference (MD) for sleep quality domains based on VSHSS between intervention and control groups, **(A)** disturbance; **(B)** effectiveness and **(C)** supplementary.

Eight studies (14, 27, 31, 32, 37, 42, 45, 49), with 606 participants (307 and 299 patients in the intervention and control group, respectively), reported data on sleep outcomes using the VSHSS scale. Of these 8 studies, only three studies (31, 32, 49), were able to combine their data and implement meta-analysis on them. The VSHSS scale is a visual scale that evaluates the three domains of sleep disturbance (Seven items), effectiveness (Five items) and supplementary sleep (Four items) with separate scoring. Each item is answered by marking the samples on a graded vector with scores varies from 0 to 100 mm (57). Lower scores in sleep disturbance and supplementary sleep and higher scores in the effectiveness of sleep domains indicate a more satisfying sleep quality (57). Three trials reported the sleep disturbance and effectiveness sleep domains (31, 32, 49). However, only two trials reported the supplementary sleep domain *via* VSHSS (31, 49). The meta-analysis demonstrated a positive effect of using eye masks and/or earplugs on domains of sleep disturbance (MD = −19.82, 95% CI= −35.54−4.11, *P* < 0.001). However, substantial heterogeneity was also observed across the studies (*I*^2^ = 95.67%. *P* < 0.001). The result showing that the average of sleep disturbance of the treatment group was 19.82 points lower than that of the control group and indicating that the interventions might be beneficial to improve sleep disturbance in critically ill patients (Figure 4A). However, no significant differences were obtained for effectiveness and supplementary sleep domain between treatment and control groups (Figures 4B,C). *P*-values of Egger and Begg tests indicated non-significant coefficient values for publication bias for sleep disturbance (Egger test: *P* = 0.067 and Begg test: *P* = 0.111), effectiveness (Egger test: *P* = 0.052 and Begg test: *P* = 0.067), and supplementary sleep (Egger test: *P* = 0.063 and Begg test: *P* = 0.296).

#### Sleep Quality Outcomes Based on PSG

Five studies (21, 25, 33, 38, 46), with 128 participants (61 and 67 subjects in the intervention and control group, respectively), reported data on sleep outcomes using PSG. Three studies were performed in simulated ICU environment among healthy individuals and reported the outcomes on the mean (SD) scale (21, 25, 38). While two other studies have been done in the ICU and reported the results at different scales (33, 46). Therefore, meta-analysis was performed in three studies that could combine data (21, 25, 38). According to the results of these studies, sleep in simulated ICU environment was shown to be significantly fragmented, with prolonged sleep latencies, frequent arousals, a reduction or absence of rapid eye movement (REM) stage of sleep, an increase in stage 2 of non-REM sleep, and a reduction or absence of deep or slow-wave stage 3 of non-REM sleep. The pooled analyses were conducted to explore the efficacy of eye masks and/or earplugs interventions on sleep quality based on PSG, as shown in Table 4. Meta-analysis findings showed that the use of eye masks and/or earplugs resulted in a significant increase in total sleep time (MD = 25.47, 95% CI = 8.05–42.90, *P* < 0.001), sleep efficiency (MD = 0.06, 95% CI = 0.01–0.1, *P* = 0.01), REM (MD = 4.66, 95% CI = 2.7–6.62, *P* < 0.001), and a significant reduction of awaking (MD = −8.40, 95% CI = −10.15−6.64, *P* < 0.001), and sleep arousals index (MD = −5.17, 95% CI = −6.58−3.75, *P* < 0.001) (Supplementary Figures 7–10).

**Table 4:** Efficacy of eye masks and/or earplugs interventions for sleep quality based on polysomnography.

**Sleep components**	**Pooled MD (95% CI)**	***P*-value**	***I*^2^ (%)**	**Egger *P*-value**	**Begg *P*-value**
Time in bed (min)	0.28 (−4.32, 4.88)	0.90	0	0.556	0.149
Total sleep time (min)	25.4 (8.05, 42.9)	<0.001^*^	0	0.310	0.065
Sleep efficiency index	0.06 (0.01, 0.10)	0.01^*^	0	0.984	0.500
REM (%)	4.66 (2.70, 6.62)	<0.001^*^	0	0.827	0.149
Stage 1 non-REM (%)	1.65 (−0.26, 3.56)	0.09	0	0.390	0.151
Stage 2 non-REM (%)	−1.85 (−4.43, 0.74)	0.16	0	0.390	0.151
Stage 3 non-REM (%)	−0.35 (−2.10, 1.41)	0.70	0	0.933	0.999
Sleep onset latency (min)	−17.61 (-45.86, 10.63)	0.22	15.9	0.208	0.149
REM latency (min)	−16.93 (−42.48, 8.62)	0.19	16.2	0.201	0.149
No. of awakenings	−8.40 (−10.15, −6.64)	<0.001^*^	0.63	0.270	0.500
Sleep arousals index	−5.17 (-6.58, −3.75)	<0.001^*^	0	0.452	0.500

* *P < 0.05 was considered as significant*.

*MD, Mean Difference; CI, Confidence Interval; REM, Rapid eye movement*.

## Discussion

In the study, the effect of using eye masks and/or earplugs on quality of sleep was investigated in critical care setting and simulated environment of intensive care. Our study systematically reviewed 36 available studies, and 21 studies were included in the meta-analyses based on various sleep quality assessment tools. The results indicated that eye masks and/or earplugs interventions might have a positive effect on the sleep quality in critically ill patients. According to the overall PSQI sleep quality score, the eye masks and/or earplugs interventions had a positive effect on the sleep quality (26, 28, 29, 39). However, no significant difference was identified for sleep components based on PSQI score (26, 28, 29). Eleven studies reported the efficacy of eye masks and/or earplugs interventions on the overall sleep quality of critically ill patients using the RCSQ, and statistical significance in meta-analyses was observed, especially with respect to sleep depth, sleep latency, number of awakenings, sleep efficiency, and sleep quality (3, 5, 24, 40, 46–48, 51–53). Based on three studies, a positive effect of using eye masks and/or earplugs on domains of sleep disturbance *via* VSHSS was observed (31, 32, 49). Three studies measured sleep variables objectively by using PSG in a simulated critical care environment (21, 25, 38). Because of these similar conditions, we used these three studies. However, the results of these studies are not generalizable and should be interpreted with caution. The pooled results for the intervention groups showed beneficial impact (*P* < 0.05) for increased sleep period, sleep efficiency, REM sleep and decreased awaking and sleep arousals index. But the results should be treated with caution because of the studies were conducted in a simulated ICU environment with healthy adults and small sample sizes.

Eight studies used various instruments to evaluate the effectiveness of eye masks and earplugs on the sleep quality of ICU patients. One author, Le Guen et al. (34), used the Medical Outcome Study Scale (MOSS) and the Spiegel Scale (SS) and confirmed a statistically significant improvement after the intervention (*P* = 0.006). A non-randomized controlled trial study by Ho et al. (50), used the Insomnia Severity Index (ISI) questionnaire and they did not find any statistical significant difference between the intervention and control groups. In an experimental study by Baghaei et al. (30), 40 eligible patients were randomly assigned to control and eye mask groups and the Leeds Sleep Evaluation Questionnaire (LSEQ) was used to assess the effect of eye masks on nighttime sleep in CCU patients. According to the findings of this study, the use of eye masks improves sleep. Other authors; Richardson et al. (13), Jones et al. (22), Van Rompaey et al. (23), Sweity et al. (43), and Chaudhary et al. (41), employed their original questionnaires, which included a varied amount of items with different content focus. Due to the significant variety or lack of further details of questionnaires, these studies were not included in the meta-analysis. However, all of them had consensus on the positive effect of using eye masks and earplugs on the subjective quality of sleep.

In a review of 11 studies by Xie et al. (58), showed that noise was the most important cause of sleep disorders in critical care setting. The most disturbing noise sources were staff conversations and alarms, especially those with high frequencies. In addition to reducing noise by earplugs, this improvement of the sleep pattern *via* using eye masks can be explained by the relation between sleep wakefulness rhythm and the light-dark cycle. In this context, it is known that in the suprachiasmatic nucleus, the connections of the retina orient the nervous system about the existence of the light, which, being absent, stimulates the secretion of melatonin through the pineal gland (59). In a number of studies (13, 22, 25), the convenience of interventions was assessed based on patients' feedback. Many subjects reported that these interventions were comfortable and tolerable, and overall the rankings show that the products were very comfortable, very helpful, and very easy to use. However, it is important to note that earplugs and eye masks are only recommended for patients who are alert enough to cooperate and agree to these measures. Despite the evidence, the use of eye masks and earplugs may be considered invasive, especially if the patient is unable to remove them without assistance.

To our knowledge, this is the first systematic review and meta-analysis to observe the efficacy of eye masks and earplugs interventions on sleep quality in critically ill patients. Consistent with previous review studies (15, 16) on the effects of eye masks and /or earplug interventions on sleep quality in intensive care patients. The difference is that there was no meta-analysis in this area based on sleep quality assessment tools. However, there still exist several limitations in our research. The main limitation of this study was that due to the heterogeneity of studies on participants' demographic and clinical characteristics, methodological limitations, as well as measures and expression of outcomes, many of them did not enter the meta-analysis. In addition, small sample size, short evaluation period, different mental and objective sleep assessment techniques, and other methodological problems, such as lack of double blindness, and the use of simulation environment were other limitations of this study. Due to these limitations, the results of these studies are not generalizable and should be interpreted with caution. In addition, further high-quality research is needed to strengthen the evidence base.

## Conclusion

According to the data presented in the study, non-invasive and low-cost sound- and light-masking interventions like as earplugs and eye masks may improve objective sleep characteristics aswell as subjective sleep experiences of patients in critical care settings.

## Data Availability Statement

The original contributions presented in the study are included in the article/Supplementary Material, further inquiries can be directed to the corresponding author.

## Author Contributions

AV-A, FR-B, and MK-F designed the study. AV-A, SA, and SM contributed to the concept of the review and meta-analysis, acquisition of data, analysis and interpretation of data, and drafting the article. All authors edited and revised manuscript, and approved final version of manuscript.

## Conflict of Interest

The authors declare that the research was conducted in the absence of any commercial or financial relationships that could be construed as a potential conflict of interest.

## Publisher's Note

All claims expressed in this article are solely those of the authors and do not necessarily represent those of their affiliated organizations, or those of the publisher, the editors and the reviewers. Any product that may be evaluated in this article, or claim that may be made by its manufacturer, is not guaranteed or endorsed by the publisher.
