# Supplementary material for: The Efficacy of Eye Masks and Earplugs Interventions for Sleep Promotion in Critically Ill Patients: A Systematic Review and Meta-Analysis
Source: Front Psychiatry. 2021 Dec 3;12:791342. doi: 10.3389/fpsyt.2021.791342 (PMC8678458; doi:10.3389/fpsyt.2021.791342)
Supplement: Supplementary file 1 [file Data_Sheet_1.docx]

**Table 1:** Methodological quality assessment for RCTs included studies using the Joanna Briggs Institute (JBI) tool

| **First Author, Year (Ref)** | **Q1** | **Q2** | **Q3** | **Q4** | **Q5** | **Q6** | **Q7** | **Q8** | **Q9** | **Q10** | **Q11** | **Q12** | **Q13** | **Final Quality Score** | **Rating** |
| --- | --- | --- | --- | --- | --- | --- | --- | --- | --- | --- | --- | --- | --- | --- | --- |
| Van Rompaey, 2008-2010 (1) | Y | NC | Y | NC | NC | NC | Y | Y | Y | Y | Y | Y | Y | 9 | Medium |
| Hu, 2009 (2) | Y | Y | Y | Y | NC | Y | Y | Y | Y | Y | Y | Y | Y | 12 | Good |
| Daneshmandi, 2010 (3) | Y | Y | No | NC | NC | Y | Y | Y | Y | Y | No | Y | Y | 9 | Medium |
| Nieseh, 2010 (4) | Y | Y | No | NC | NC | NC | Y | Y | Y | Y | No | Y | Y | 8 | Medium |
| Neyse, 2011, Iran (5) | Y | Y | No | NC | NC | NC | Y | Y | Y | Y | No | Y | Y | 8 | Medium |
| Yazdannik, 2012 (6) | Y | Y | No | NC | NC | NC | Y | Y | Y | Y | NC | Y | Y | 8 | Medium |
| Demoule, 2011-2013 (7) | Y | Y | Y | Y | NC | NC | Y | Y | Y | Y | NC | Y | Y | 9 | Medium |
| Guen, 2013 (8) | Y | Y | No | NC | NC | Y | Y | Y | Y | Y | No | Y | Y | 9 | Medium |
| Babaii, 2013 (9) | Y | Y | Y | Y | NC | NC | Y | Y | Y | Y | NC | Y | Y | 10 | Good |
| Bajwa, 2014 (10) | Y | Y | Y | Y | NC | NC | Y | Y | Y | Y | Y | Y | Y | 11 | Good |
| Huang, 2014 (11) | Y | Y | NO | Y | NC | NC | Y | Y | Y | Y | Y | Y | Y | 10 | Good |
| Cheraghi, 2104 (12) | Y | Y | No | NC | NC | Y | Y | Y | Y | Y | No | Y | Y | 9 | Medium |
| Litton, 2015-2016 (13) | Y | Y | Y | NA | NA | NA | Y | Y | Y | Y | Y | Y | Y | 10 | Good |
| Chaudhary, 2016 (14) | Y | Y | Y | NC | NC | NC | Y | Y | Y | Y | Y | Y | Y | 10 | Good |
| Sharafi, 2016 (15) | Y | Y | Y | Y | NC | NC | Y | Y | Y | Y | Y | Y | Y | 11 | Good |
| Sweity, 2017 (16) | Y | Y | Y | Y | NC | NC | Y | Y | Y | Y | NC | Y | Y | 10 | Good |
| Arttawejkul, 2017-2018 (17) | NC | NC | Y | NC | NC | Y | Y | Y | Y | Y | Y | Y | No | 8 | Medium |
| Obanor, 2018 (18) | Y | Y | N | NC | NC | NC | Y | Y | Y | Y | Y | Y | Y | 9 | Medium |
| Lakeh, 2018 (19) | Y | Y | Y | NC | NC | NC | Y | Y | Y | Y | Y | Y | Y | 10 | Good |
| Ho, 2018-2019 (20) | Y | Y | Y | NC | NC | NC | Y | Y | Y | Y | Y | Y | Y | 10 | Good |
| Obanor, 2018-2019 (21) | Y | Y | Y | NC | NC | NC | Y | Y | Y | Y | Y | Y | Y | 10 | Good |
| Mahran, 2107 (22) | Y | Y | Y | Y | NC | NC | Y | Y | Y | Y | Y | Y | Y | 11 | Good |
| Leong, 2018-2019 (23) | Y | Y | Y | Y | NC | NC | Y | Y | Y | Y | Y | Y | Y | 11 | Good |

Y: Yes, N: No, NC: Not clear, NA: Not applicable. (Q1. Was true randomization used for assignment of participants to treatment groups? Q2. Was allocation to treatment groups concealed? Q3. Were treatment groups similar at the baseline? Q4. Were participants blind to treatment assignment? Q5. Were those delivering treatment blind to treatment assignment? Q6. Were outcomes assessors blind to treatment assignment? Q7. Were treatment groups treated identically other than the intervention of interest? Q8. Was follow up complete and if not, were differences between groups in terms of their follow up adequately described and analyzed? Q9. Were participants analyzed in the groups to which they were randomized? Q10. Were outcomes measured in the same way for treatment groups? Q11. Were outcomes measured in a reliable way? Q12. Was appropriate statistical analysis used? Q13. Was the trial design appropriate, and any deviations from the standard RCT design (individual randomization, parallel groups) accounted for in the conduct and analysis of the trial? Rating—Good, Medium or Poor, Good = (≥10 yes); Medium = (6-9 yes), Poor = (≤5 yes).

**Table 2:** Methodological quality assessment for Quasi-experimental included studies using the Joanna Briggs Institute (JBI) tool

| **First Author, Year (Ref)** | **Q1** | **Q2** | **Q3** | **Q4** | **Q5** | **Q6** | **Q7** | **Q8** | **Q9** | **Final Quality Score** | **Rating** |
| --- | --- | --- | --- | --- | --- | --- | --- | --- | --- | --- | --- |
| Wallace, 1999 (24) | Y | NC | Y | Y | Y | Y | Y | Y | Y | 8 | Good |
| Richardson, 2007 (25) | Y | Y | Y | Y | Y | NA | NC | NC | Y | 6 | Medium |
| Scotto, 2009 (26) | Y | N | Y | Y | N | Y | NC | NC | Y | 5 | Medium |
| Jones, 2008-2009 (27) | Y | Y | Y | Y | NA | NA | NC | Y | Y | 6 | Medium |
| Hu, 2010 (28) | Y | Y | Y | Y | Y | Y | NC | Y | Y | 8 | Good |
| Ryu, 2010 (29) | Y | Y | Y | Y | Y | Y | NC | Y | Y | 8 | Good |
| Baghaei, 2011-2012 (30) | Y | N | Y | Y | N | NC | Y | Y | Y | 6 | Medium |
| Mashayekhi, 2012 (31) | Y | N | Y | Y | N | Y | NC | Y | Y | 6 | Medium |
| Kamdar, 2013 (32) | Y | Y | Y | Y | N | NC | NC | Y | Y | 6 | Medium |
| Bani Younis, 2017 (33) | Y | Y | Y | Y | Y | Y | Y | Y | Y | 9 | Good |
| Dobing, 2017 (34) | Y | Y | Y | Y | N | Y | Y | Y | Y | 8 | Good |
| Koçak, 2017-2018 (35) | Y | Y | Y | Y | N | NC | Y | Y | Y | 7 | Good |

Y: Yes, N: No, NC: Not clear, NA: Not applicable. (Q1. Is it clear in the study what is the ‘cause’ and what is the ‘effect’ (i.e. there is no confusion about which variable comes first)? Q2. Were the participants included in any comparisons similar? Q3. Were the participants included in any comparisons receiving similar treatment/care, other than the exposure or intervention of interest? Q4. Was there a control group? Q5. Were there multiple measurements of the outcome both pre and post the intervention/exposure? Q6. Was follow up complete and if not, were differences between groups in terms of their follow up adequately described and analyzed? Q7. Were the outcomes of participants included in any comparisons measured in the same way? Q8. Were outcomes measured in a reliable way? Q9. Was appropriate statistical analysis used? Rating—Good, Medium or Poor, Good = (≥7 yes), Medium= (4-6) and Poor = (≤3 yes)

| **Web of science:** ((**TOPIC:** (((((((((((((((sleep OR "sleep disorder") OR "sleep deprivation") OR "sleep apnea") OR hypersomnolence) OR insomnia) OR dyssomnia) OR "Sleep latency") OR rem) OR "rapid eye movement") OR "Non REM") OR "Non rapid eye movement") OR "Sleep stage") OR "Sleep quality") OR "Sleep time") OR "Sleep wake disorder" OR "eye mask" OR "earplugs") *AND* **TOPIC:** ((((("Intensive care" OR "Critical care") OR icu) OR ccu) OR intensive) OR critical)) *AND* **TOPIC:** (disorder OR disturbance))  **Scopus:** ( TITLE-ABS-KEY ( *sleep*  OR  *"sleep disorder"*  OR  *"sleep deprivation"*  OR  *"sleep apnea"*  OR  *hypersomnolence*  OR  *insomnia*  OR  *dyssomnias*  OR  *"Sleep latency"*  OR  *rem*  OR  *"rapid eye movement"*  OR  *"Non REM"*  OR  *"Non rapid eye movement"*  OR  *"Sleep stage"*  OR  *"Sleep quality"*  OR  *"Sleep time"* )  OR  TITLE-ABS-KEY ( *"Sleep wake disorder"* OR "eye mask" OR "earplugs")  AND  TITLE-ABS-KEY ( *"Intensive care"*  OR  *"Critical care"*  OR  *icu*  OR  *ccu*  OR  *intensive*  OR  *critical* )  AND  TITLE-ABS-KEY ( *disorder*  OR  *disturbance* ) )  **Pubmed:** (((sleep [Title/Abstract] OR "sleep disorder" [Title/Abstract] OR "sleep deprivation" [Title/Abstract] OR "sleep apnea" [Title/Abstract] OR hypersomnolence [Title/Abstract] OR insomnia [Title/Abstract] OR dyssomnias [Title/Abstract] OR "Sleep latency" [Title/Abstract] OR rem [Title/Abstract] OR "rapid eye movement" [Title/Abstract] OR "Non REM" [Title/Abstract] OR "Non rapid eye movement" [Title/Abstract] OR "Sleep stage" [Title/Abstract] OR "Sleep quality" [Title/Abstract] OR "Sleep time"[Title/Abstract] OR "Sleep wake disorder"[Title/Abstract] OR "eye mask" OR "earplugs"[Title/Abstract])) AND ("Intensive care" [Title/Abstract] OR "Critical care" [Title/Abstract] OR icu [Title/Abstract] OR ccu [Title/Abstract] OR intensive [Title/Abstract] OR critical[Title/Abstract])) AND (disorder [Title/Abstract] OR disturbance[Title/Abstract])  **Proquest:** ab(sleep OR "sleep disorder" OR "sleep deprivation" OR "sleep apnea" OR hypersomnolence OR insomnia OR dyssomnias OR "Sleep latency" OR rem OR "rapid eye movement" OR "Non REM" OR "Non rapid eye movement" OR "Sleep stage" OR "Sleep quality" OR "Sleep time" OR "Sleep wake disorder" OR "eye mask" OR "earplugs")  AND ab("Intensive care" OR "Critical care" OR icu OR ccu OR intensive OR critical) AND ab(disorder OR disturbance) |
| --- |

**Table 3:** Search strategy

**Table 4: Demographic Characteristics of participants**

| **First Author, Year, country (Ref)** | **Sample size** | | **Age (mean)** | | | **Gender, male (%)** | | |
| --- | --- | --- | --- | --- | --- | --- | --- | --- |
|  | I | C | I | C | T | I | C | T |
| Wallace, 1999, US (24) | 3 | 3 | NR | NR | NR | NR | NR |  |
| Richardson, 2007, UK (25) | 34 | 28 | NR | NR | NR | NR | NR | M: 44 (69)  F: 20 (31) |
| Scotto, 2009, US (26) | 49 | 39 | 63.7 | 62.5 | NR | 27 (55.2) | 26 (66.7) | M: 53 (60.3)  F: 35 (39.7) |
| Jones, 2008-2009, UK (27) | 50 | 50 | 58.07 | 56.34 | NR | 27 (54) | 30 (60) | M: 57 (57)  F: 53 (53) |
| Van Rompaey, 2008-2010, Belgium (1) | 69 | 67 | 57 | 62 | NR | 68.1% | 64.2% | - |
| Hu, 2009, China (2) | 20 | 25 | 56.6 | 56.8 | NR | 11 (55) | 16 (64) | M: 27 (60)  F: 18 (40) |
| Hu, 2010, China (28) | 7 | 7 | NR | NR | 31.07 | NR | NR | M: 6 (42.8)  F: 8 (57.2) |
| Daneshmandi, 2010, Iran (3) | 30 | 30 | NR | NR | 55.9 | 16 (53.3) | 13 (43.3) | M: 29 (48.3)  F: 31 (51.7) |
| Ryu, 2010, China (29) | 29 | 29 | NR | NR | NR | 19 (32.8) | 19 (32.8) | M: 38 (65.5)  F: 20 (34.5) |
| Nieseh, 2010 , Iran (4) | 30 | 30 | NR | NR | 54.9 | NR | NR | M: 29 (48.3)  F: 31 (51.7) |
| Neyse, 2011, Iran (5) | 30 | 30 | NR | NR | 54.9 | NR | NR | M: 29 (48.3)  F: 31 (51.7) |
| Baghaei, 2011-2012, Iran (30) | 20 | 20 | 59.80 | 60.20 | NR | 11 (55) | 14 (70) | M: 25 (62.5)  F: 15 (37.5) |
| Mashayekhi, 2012, Iran (31) | 30 | 30 | NR | NR | 58 | NR | NR | M: 34 (56.7)  F: 26 (43.3) |
| Yazdannik, 2012, Iran (6) | 25 | 25 | NR | NR | NR | NR | NR | M: 23 (46)  F: 27 (54) |
| Demoule, 2011-2013, France (7) | 23 | 28 | 64 | 65 | NR | 20 (67) | 18 (58) | M: 38 (74.5)  F: 13 (25.5) |
| Guen, 2013, France, (8) | 20 | 21 | 62 | 59 | NR | 15 (75) | 19 (90.4) | M: 34 (83)  F: 7 (17) |
| Babaii, 2013 Iran (9) | 30 | 30 | 61.4 | 63.9 | NR | 16 (53.3) | 9 (30) | M: 25 (41.7)  F: 35 (58.3) |
| Kamdar, 2013, US (32) | 110 | 185 | 54* | 53* | NR | NR | NR | M: 225 (76.3)  F: 70 (23.7) |
| Bajwa, 2014, India (10) | 50 | 50 | NR | NR | 58.7 | 25 (50) | 26 (52) | M: 51 (51)  F: 49 (49) |
| Huang, 2014, China (11) | 20 | 20 | NR | NR | 40.1 | NR | NR | M: 20 (50)  F: 20 (50) |
| Cheraghi, 2104, Iran (12) | 36 | 36 | 59.39 | 62.08 | NR | 22 (61.1) | 24 (66.7) | M: 46 (63.9)  F: 26 (36.1) |
| Litton, 2015-2016, Australia (13) | 20 | 20 | 70* | 66* | NR | 14 (70) | 17 (85) | M: 31 (77.5)  F: 9 (22.5) |
| Chaudhary, 2016, India (14) | 30 | 30 | 49.7 | 52.7 | 51.2 | 12 (40) | 19 (63.3) | M: 31 (51.7)  F: 29 (48.3) |
| Sharafi, 2016, Iran (15) | 36 | 37 | 49.7 | 54 | NR | 22 (61.1) | 27 (73) | M: 49 (67.1)  F: 24 (32.9) |
| Sweity, 2017, UK (16) | 109 | 97 | 48.4 | 55.3 | NR | 39 (35.8) | 46 (47.4) | M: (51.7)  F: (48.3) |
| **First Author, Year, country (Ref)** | **Sample size** | | **Age (mean)** | | | **Gender, male (%)** | | |
|  | I | C | I | C | T | I | C | T |
| Bani Younis, 2017, Jordan, (33) | 52 | 51 | 51.18 | 56.18 | NR | NR | NR | NR |
| Dobing, 2017, Canada, (34) | 40 | 41 | 61.4 | 58.6 | 60 | 23 (57.5) | 27 (65.8) | M: 50 (61.7)  F: 31 (38.3) |
| Arttawejkul, 2017-2018, India (17) | 8 | 9 | 67 | 76 | NR | 6 (75) | 5 (56) | M: 11 (64.7)  F: 6 (35.3) |
| Baghaie Lakeh, 2018, Iran (19) | 48 | 48 | 59.81 | 61.89 | NR | 45.8% | 39.6% | NR |
| Obanor, 2018-2019, US (21) | 44 | 43 | 51.4 | 50.7 | NR | NR | NR | NR |
| Mahran, 2107, Eygept (22) | 31 | 35 | 48.03 | 46.91 | NR | 23 (74) | 11 (31) | M: 34 (51.5)  F: 32 (48.5) |
| Leong, 2018-2019, Singapore (23) | 48 | 45 | 60 | 67 | NR | 25 (52.1) | 26 (57.7) | M: 51 (54.8)  F: 42 (45.2) |

I: intervention, C: control, T: total, *Median

**References**

1. Van Rompaey B, Elseviers MM, Van Drom W, Fromont V, Jorens PG. The effect of earplugs during the night on the onset of delirium and sleep perception: a randomized controlled trial in intensive care patients. *Critical care (London, England)*. (2012) 16:R73. doi: 10.1186/cc11330

2. Hu RF, Jiang XY, Hegadoren KM, Zhang YH. Effects of earplugs and eye masks combined with relaxing music on sleep, melatonin and cortisol levels in ICU patients: a randomized controlled trial. *Critical care (London, England)*. (2015) 19:115. doi: 10.1186/s13054-015-0855-3

3. Daneshmandi M, Neiseh F, SadeghiShermeh M, Ebadi A. Effect of eye mask on sleep quality in patients with acute coronary syndrome. *Journal of caring sciences*. (2012) 1:135-43. doi: 10.5681/jcs.2012.020

4. Neiseh F, Daneshmandi M, Sadeghi Sharme Mahdi, Ebadi Abbas. The effects of ear and eue protect device applicatio on quality of sleep of CCU patients with acute coronary syndrome (Hospital Military). *Journal of Millitary Psychology (JMP)*. (2011) 2:-. doi:

5. Neiseh F, Daneshmandi M, Sadeghi Sharme Mahdi, Ebadi Abbas. The effect of earplugs on sleep quality in patients with acute coronary syndrome. *IJCCN*. (2011) 4:-. doi:

6. Yazdannik AR, Zareie A, Hasanpour M, Kashefi P. The effect of earplugs and eye mask on patients' perceived sleep quality in intensive care unit. *Iranian journal of nursing and midwifery research*. (2014) 19:673-8. doi:

7. Demoule A, Carreira S, Lavault S, Pallanca O, Morawiec E, Mayaux J, et al. Impact of earplugs and eye mask on sleep in critically ill patients: a prospective randomized study. *Critical Care*. (2017) 21. doi: <http://dx.doi.org/10.1186/s13054-017-1865-0>

8. Le Guen M, Nicolas-Robin A, Lebard C, Arnulf I, Langeron O. Earplugs and eye masks vs routine care prevent sleep impairment in post-anaesthesia care unit: a randomized study. *British journal of anaesthesia*. (2014) 112:89-95. doi: 10.1093/bja/aet304

9. Babaii A, Adib-Hajbaghery M, Hajibagheri A. Effect of Using Eye Mask on Sleep Quality in Cardiac Patients: A Randomized Controlled Trial. *Nursing and midwifery studies*. (2015) 4:e28332. doi: 10.17795/nmsjournal28332

10. Bajwa N, Saini P, Kaur H, Kalra S, Kaur J. Effect of ear plugs and eye mask on sleep among ICU patients: a randomized control trial. *Int J Curr Res*. (2015) 7: 741–5. doi:

11. Huang HW, Zheng BL, Jiang L, Lin ZT, Zhang GB, Shen L, et al. Effect of oral melatonin and wearing earplugs and eye masks on nocturnal sleep in healthy subjects in a simulated intensive care unit environment: which might be a more promising strategy for ICU sleep deprivation? *Critical care (London, England)*. (2015) 19:124. doi: 10.1186/s13054-015-0842-8

12. cheraghi ma, akbari k, Bahramnezhad f, haghani h. The Effect of Earplug on the Sleep of Patients Hospitalized in the Coronary Care Unit. *complementary Medicine Journal*. (2018) 8:2293-303. doi:

13. Litton E, Elliott R, Ferrier J, Webb SAR. Quality sleep using earplugs in the intensive care unit: the QUIET pilot randomised controlled trial. *Critical care and resuscitation : journal of the Australasian Academy of Critical Care Medicine*. (2017) 19:128-33. doi:

14. Chaudhary A, Kumari V, Neetu N. Sleep Promotion among Critically Ill Patients: Earplugs/Eye Mask versus Ocean Sound-A Randomized Controlled Trial Study. *Critical care research and practice*. (2020) 2020:8898172. doi: 10.1155/2020/8898172

15. Sharafi S, Hajiabadi F, Razi M, Bahrami M. The Effect of Simultaneously Using Earplug and Eye mask on Quality of Sleep in Intensive Care Unit Patients: A Randomized Clinical Trial Study. *Journal of Critical Care Nursing*. (2018) 11:27-34. doi:

16. Sweity S, Finlay A, Lees C, Monk A, Sherpa T, Wade D. SleepSure: a pilot randomized-controlled trial to assess the effects of eye masks and earplugs on the quality of sleep for patients in hospital. *Clinical rehabilitation*. (2019) 33:253-61. doi: 10.1177/0269215518806041

17. Arttawejkul P, Reutrakul S, Muntham D, Chirakalwasan N. Effect of Nighttime Earplugs and Eye Masks on Sleep Quality in Intensive Care Unit Patients. *Indian journal of critical care medicine : peer-reviewed, official publication of Indian Society of Critical Care Medicine*. (2020) 24:6-10. doi: 10.5005/jp-journals-10071-23321

18. Obanor O, McBroom M, Elia J, Sasaki J, Murphy K, Chalk S, et al. Preliminary analysis of impact of earplugs and eye masks on sleep quality for patients in a SICU. *Critical care medicine*. (2019) 47. doi:

19. Lakeh MB, Lakeh MB, Mohammadi TK, Leyli EKN. The Effect of Use of Earplugs on Sleep Quality in Coronary Care Units Patients. *J Holistic Nurs Midwifery*. (2018) 28:93-100. doi: 10.29252/hnmj.28.2.93

20. Ho AT, Waldhorn RE, Joshi A, Thapaliya S, Hunt T, Diamond M, et al. A Pilot Non-Randomized Control Trial of Providing Quiet Time, Ear Plugs, Eye Masks, Reducing Noise and Light in Improving Sleep for Patients in General Medical Wards. *Am J Respir Crit Care Med*. (2019) 199. doi:

21. Obanor OO, McBroom MM, Elia JM, Ahmed F, Sasaki JD, Murphy KM, et al. The Impact of Earplugs and Eye Masks on Sleep Quality in Surgical ICU Patients at Risk for Frequent Awakenings. *Critical care medicine*. (2021). doi: 10.1097/ccm.0000000000005031

22. Mahran GS, Leach MJ, Abbas MS, Abbas AM, Ghoneim AM. Effect of Eye Masks on Pain and Sleep Quality in Patients Undergoing Cardiac Surgery: A Randomized Controlled Trial. *Critical care nurse*. (2020) 40:27-35. doi: 10.4037/ccn2020709

23. Leong RW, Davies LJ, Fook-Chong S, Ng SY, Lee YL. Effect of the use of earplugs and eye masks on the quality of sleep after major abdominal surgery: a randomised controlled trial. *Anaesthesia*. (2021). doi: 10.1111/anae.15468

24. Wallace CJ, Robins J, Alvord LS, Walker JM. The effect of earplugs on sleep measures during exposure to simulated intensive care unit noise. *Am J Crit Care*. (1999) 8:210-9. doi: 10.4037/ajcc1999.8.4.210

25. Richardson A, Allsop M, Coghill E, Turnock C. Earplugs and eye masks: do they improve critical care patients' sleep? *Nursing in critical care*. (2007) 12:278-86. doi: 10.1111/j.1478-5153.2007.00243.x

26. Scotto CJ, McClusky C, Spillan S, Kimmel J. Earplugs improve patients' subjective experience of sleep in critical care. *Nursing in critical care*. (2009) 14:180-4. doi: 10.1111/j.1478-5153.2009.00344.x

27. Jones C, Dawson D. Eye masks and earplugs improve patient's perception of sleep. *Nursing in critical care*. (2012) 17:247-54. doi: 10.1111/j.1478-5153.2012.00501.x

28. Hu RF, Jiang XY, Zeng YM, Chen XY, Zhang YH. Effects of earplugs and eye masks on nocturnal sleep, melatonin and cortisol in a simulated intensive care unit environment. *Critical care (London, England)*. (2010) 14:R66. doi: 10.1186/cc8965

29. Ryu MJ, Park JS, Park H. Effect of sleep-inducing music on sleep in persons with percutaneous transluminal coronary angiography in the cardiac care unit. *Journal of clinical nursing*. (2012) 21:728-35. doi: 10.1111/j.1365-2702.2011.03876.x

30. Baghaei R, Feizi A, Ghaderi C. The effect of eye masks on nocturnal sleep in patients hospitalized in the Intensive Cardiac Care Units. *Nursing and Midwifery Journal*. (2014) 12:800-6. doi:

31. Mashayekhi F, Arab M, Pilevarzadeh M, Amiri M, Rafiei H. The effect of eye mask on sleep quality in patients of coronary care unit. *Sleep Sci*. (2013) 6:108-11. doi:

32. Kamdar BB, King LM, Collop NA, Sakamuri S, Colantuoni E, Neufeld KJ, et al. The effect of a quality improvement intervention on perceived sleep quality and cognition in a medical ICU. *Critical care medicine*. (2013) 41:800-9. doi: 10.1097/CCM.0b013e3182746442

33. Bani Younis MK, Hayajneh FA, Alduraidi H. Effectiveness of using eye mask and earplugs on sleep length and quality among intensive care patients: A quasi-experimental study. *International journal of nursing practice*. (2019) 25:e12740. doi: 10.1111/ijn.12740

34. Dobing S, Dey A, McAlister F, Ringrose J. Non-pharmacologic interventions to improve sleep of medicine inpatients: a controlled study. *Journal of Community Hospital Internal Medicine Perspectives*. (2017) 7:287-95. doi: <http://dx.doi.org/10.1080/20009666.2017.1379845>

35. Kocak AT, Arslan S. The Effect of Using Eye Masks and Earplugs on Intensive Care Patients Quality of Sleep and Vital Signs. *J Neurosci Nurs*. (2021) 53:29-33. doi: 10.1097/jnn.0000000000000562
