# Supplementary material for: The Efficacy of Eye Masks and Earplugs Interventions for Sleep Promotion in Critically Ill Patients: A Systematic Review and Meta-Analysis
Source: Front Psychiatry. 2021 Dec 3;12:791342. doi: 10.3389/fpsyt.2021.791342 (PMC8678458; doi:10.3389/fpsyt.2021.791342)
Supplement: Supplementary file 2 [file Data_Sheet_2.docx]

| **A**  **** |
| --- |
| **B**  **** |
| **C**  **** |

Fig.1. Forest plot of mean difference (MD) for sleep quality components based on PSQI, (A) sleep quality, (B) sleep latency, and (C) sleep duration

| **A**  **** |
| --- |
| **B**  **** |
| **C**  **** |

Fig.2. Forest plot of mean difference (MD) for sleep quality components based on PSQI, (A) habitual sleep efficiency, (B) sleep disturbance, and (C) use of sleeping medications

| **A**  **** |
| --- |

Fig.3. Forest plot of mean difference (MD) for sleep quality components based on PSQI, (A) daytime dysfunction

| **A**  **** |
| --- |
| **B**  **** |

Fig.4. Forest plot of mean difference (MD) for sleep quality components based on RCSQ, (A) sleep depth, and (B) sleep latency

| **A**  **** |
| --- |
| **B**  **** |

Fig.5. Forest plot of mean difference (MD) for sleep quality components based on RCSQ (A), number of awakening, and (B) sleep efficiency

| **A**  **** |
| --- |

Fig.6. Forest plot of mean difference (MD) for sleep quality components based on RCSQ, (A) sleep quality

| **A**   |
| --- |
| **B**  **** |
| **C**  **** |

Fig.7. Forest plot of mean difference (MD) for sleep quality components based on polysomnography, (A) time in bed, (B) total sleep time, and (C) sleep efficiency index

| **A**  **** |
| --- |
| **B**   |
| **C**  **** |

Fig.8. Forest plot of mean difference (MD) for sleep quality components based on polysomnography, (A) REM, (B) stage 1 non-REM, and (C) stage 2 non-REM

| **A**   |
| --- |
| **B**  **** |
| **C**   |

Fig.9. Forest plot of mean difference (MD) for sleep quality components based on polysomnography, (A) stage 3 non-REM, (B) sleep onset latency, and (C) REM latency

| **A**  **** |
| --- |
| **B**  **** |

Fig.10. Forest plot of mean difference (MD) for sleep quality components based on polysomnography, (A) No. of awakenings, and (B) sleep arousals index
